# Exploring the influence of gut microbiota metabolites on vitiligo through the gut-skin axis

**DOI:** 10.3389/fmicb.2025.1566267

**Published:** 2025-07-09

**Authors:** Chuanjian Yuan, Lyuye Liu, Duorong Zeng, Jinxiang Yuan, Liyuan Guo, Junling Zhang

**Affiliations:** ^1^Graduate School, Tianjin University of Traditional Chinese Medicine, Tianjin, China; ^2^Department of Dermatology, Affiliated Hospital of Tianjin Academy of Traditional Chinese Medicine, Tianjin, China

**Keywords:** vitiligo, gut microbiota, short-chain fatty acids, secondary bile acids, tryptophan

## Abstract

Vitiligo is an autoimmune skin disease with a complex pathogenesis closely linked to immune imbalance and oxidative stress. Currently, comprehensive curative treatments and effective relapse prevention strategies are lacking. Recently, the “gut-skin axis” hypothesis has offered new insights into the pathological mechanisms of vitiligo. Studies indicate that gut microbiota and their metabolic products significantly affect disease progression by regulating immune homeostasis and inflammatory responses in the host. This review systematically examines the effects of short-chain fatty acids, secondary bile acids, and tryptophan metabolites on the human immune system and the inflammatory milieu, and their direct impact on melanocytes. Furthermore, considering the reduced diversity of gut microbiota in individuals with vitiligo, this article also evaluates methods including probiotic intervention, the Mediterranean diet, and fecal microbiota transplantation, which may emerge as potential therapeutic strategies for vitiligo by restoring microbiota balance. Future multidimensional therapeutic strategies that target gut microbiota metabolites show promise for pioneering innovative approaches in vitiligo management.

## 1 Introduction

Vitiligo is an autoimmune disorder characterized by the progressive loss of melanocytes (MCs) and resulting skin depigmentation (Utama et al., 2024). Damage to MCs is influenced by various factors, such as genetic susceptibility, autoimmunity, and oxidative stress (Iwanowski et al., 2023). Immune responses in MCs can be triggered by endogenous or exogenous factors, including damage-associated molecular patterns (DAMPs) (Migayron et al., 2020). Current treatments for vitiligo (including topical agents, phototherapy, and immunosuppressants) primarily aim to suppress immune responses or promote localized repigmentation, yet they generally fail to completely halt disease progression or prevent recurrence. Notably, some patients may experience even more extensive depigmentation following treatment discontinuation. Furthermore, long-term use of corticosteroids or immunosuppressive agents may lead to adverse effects such as cutaneous atrophy and increased infection risks, while surgical interventions like autologous epidermal transplantation present traumatic risks and prove unsuitable for patients with extensive lesions. Collectively, these therapeutic approaches demonstrate unstable efficacy due to their insufficient targeting of vitiligo’s underlying pathogenic mechanisms. The intractable nature and high recurrence rate of vitiligo reflect the delicate balance of immune system homeostasis in affected individuals. The gut microenvironment is essential for immune system homeostasis, with the gut microbiota influencing skin health through strain transfer, metabolite exchange, and immune regulation (Sinha et al., 2021; Gao et al., 2023). Recent studies based on the “gut-skin axis” hypothesis suggest that gut microbiota and their metabolites, including short-chain fatty acids (SCFAs), tryptophan(Trp), and secondary bile acids (SBAs), play a vital role in regulating immune responses, inflammation, and metabolism (Mostafavi Abdolmaleky and Zhou, 2024; Xiao et al., 2023). The gut microbiota and its metabolites effectively maintain the intestinal barrier and regulate disease progression in skin disorders like psoriasis and atopic dermatitis (Li et al., 2024; Zhang et al., 2024; Pessôa et al., 2023). However, there is a current lack of a comprehensive review concerning the impact of gut microbiota and its metabolites on vitiligo. Consequently, this review examines the influence of gut microbiota and its metabolites on the host’s immune system and inflammation via the gut-skin axis, to identify novel therapeutic strategies for the clinical management of vitiligo.

The gut and skin both possess extensive neural and vascular networks that perform immunoregulatory and neuroendocrine functions. Sharing a common embryonic origin (the endoderm), both the gut and skin host diverse symbiotic microbial communities with significant functional correlations (Salem et al., 2018). Numerous studies provided evidence for a profound bidirectional link between gastrointestinal health and skin homeostasis through modification of the immune system (De Pessemier et al., 2021). Imbalances in the gut microbiota can disrupt mucosal immune tolerance, adversely affecting skin health (Chen et al., 2024). Similarly, skin damage can disrupt gut homeostasis and alter its microbiome (Dokoshi et al., 2024). The high comorbidity of inflammatory bowel diseases in patients with clinical vitiligo underscores the significant correlation between gut and skin health (Hadi et al., 2020). Studies have revealed significant differences in the gut microbial community’s structure and function between vitiligo patients and healthy individuals (Luan et al., 2023; Bzioueche et al., 2021; Wu et al., 2023; Ganju et al., 2016; Ni et al., 2020). Research using vitiligo mouse models suggests that antibiotics may reduce Clostridium populations in the gut, leading to the loss of skin melanocytes (Dellacecca et al., 2020). The mechanism by which the gut-skin axis influences vitiligo is closely related to gut microbial metabolites’ actions (Zhang et al., 2024). Extensive crosstalk between gut microorganisms and host-microbiota co-metabolism allows metabolite measurements to directly reflect the host-microbiota system (Krautkramer et al., 2021). These metabolites, especially SCFAs, SBAs, and Trp, regulate immune system homeostasis and the physiological functions of various organs (Gasaly et al., 2021). However, the influence of gut metabolites on vitiligo pathogenesis is not fully understood.

This article explores the synthesis, function, and signaling mechanisms of gut microbiota metabolic products, with a focus on their impact on the immune system and inflammatory processes through the gut-skin axis, particularly the relationship between major metabolites and vitiligo. The aim is to elucidate the mechanisms by which gut microbiota metabolic products influence vitiligo, and to establish a theoretical basis for their application in the clinical management of the disease.

### 1.1 The main pathophysiological mechanisms of vitiligo

**Figure 1:**
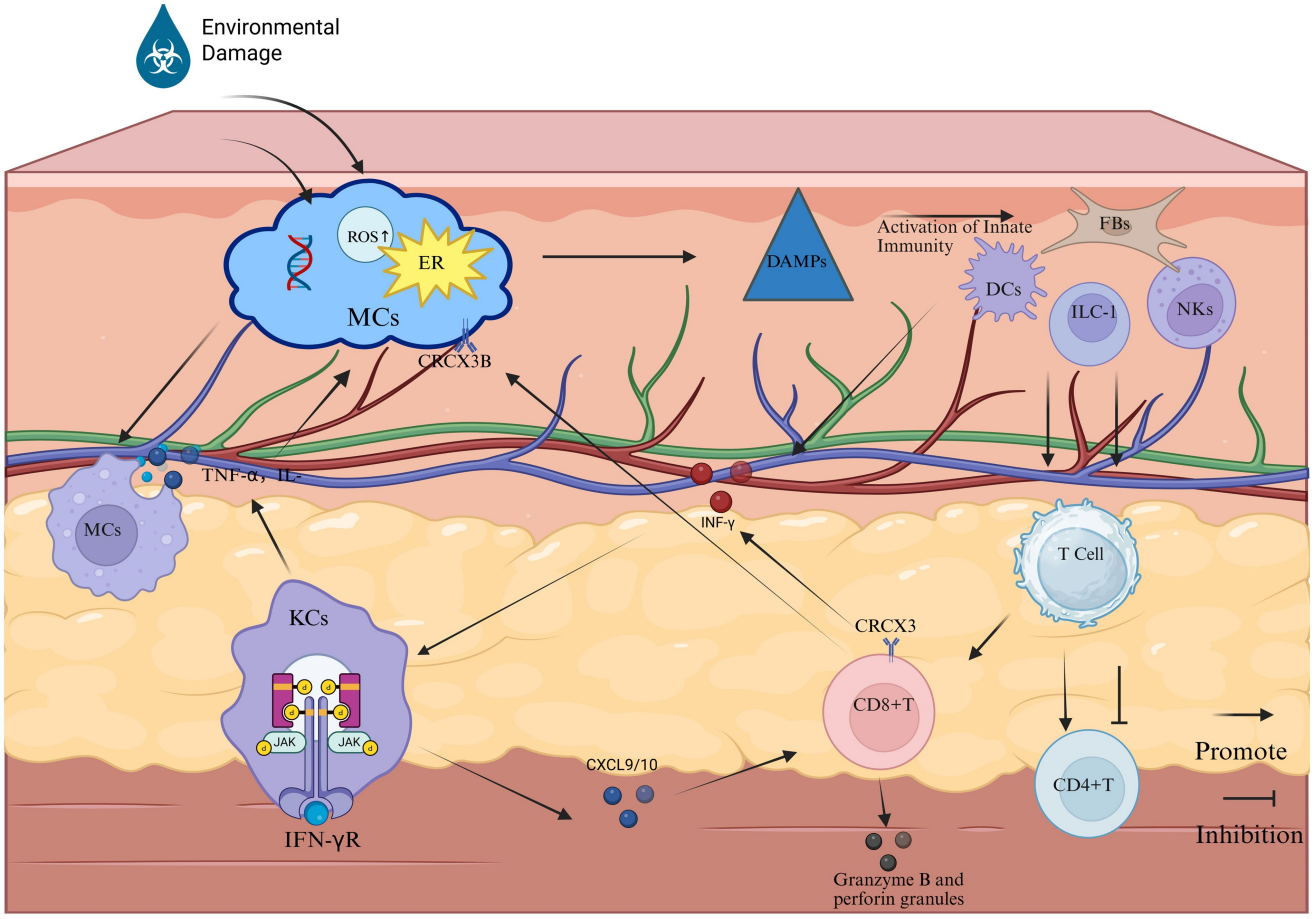
The primary pathological process of vitiligo (Created in https://BioRender.com). Phase 1: Melanocyte stress response: Stressed melanocytes manifest pathophysiological alterations characterized by endoplasmic reticulum (ER) stress, elevated ROS, and DNA damage. These cellular distress signals are recognized by cutaneous innate immune sentinels—DCs, NKs cells, ILC1s, and FBs—which subsequently undergo activation through pattern recognition receptor signaling. Phase 2: Innate immune activation: This immunogenic cascade triggers the JAK–STAT signaling axis, inducing keratinocytes to overexpress chemokines CXCL9 and CXCL10. These IFN-*γ*-inducible chemokines mediate CD8 + T cell recruitment to the dermo-epidermal junction via CXCR3 receptor ligation. Phase 3: Adaptive Immune Execution: Cytotoxic Mechanisms: Infiltrating CD8 + T lymphocytes release IFN-γ and cytolytic mediators (perforin, granzyme B), directly inducing melanocyte apoptosis. IFN-γ amplifies MHC class I expression on residual melanocytes, enhancing autoimmune targeting. Macrophage-Mediated Inflammation: Tissue-resident macrophages phagocytize apoptotic melanocytes, subsequently secreting pro-inflammatory cytokines (TNF-α, IL-6, IL-1β). These cytokines perpetuate local inflammation, disrupt melanocyte stem cell niches, and inhibit pigment regeneration. Pathogenic Feedback Loop: Sustained cytokine release (particularly IFN-γ and TNF-α) reactivates keratinocyte JAK–STAT signaling, establishing a self-reinforcing inflammatory circuit that drives progressive depigmentation.

In vitiligo, oxidative stress and immune dysregulation are significant contributors to disease onset in genetically predisposed individuals. Oxidative stress, characterized by an excessive production of reactive molecules such as reactive oxygen species (ROS) and hydrogen peroxide (H_2_O_2_), creates a pro-inflammatory microenvironment in affected tissues (Białczyk et al., 2023). This pro-inflammatory environment may activate innate immune cells, including dendritic cells (DCs) and natural killer (NKs) cells. These cells play a central role in the inflammatory response by releasing pro-inflammatory cytokines such as heat shock protein 70 (HSP70i), interleukins (e.g., IL-6, IL-17, IL-23, IL-27), and tumor necrosis factor-alpha (TNF-*α*), thereby exacerbating inflammation (Seneschal et al., 2021; Xie et al., 2023; Mukhatayev and Le Poole, 2024; Hayran et al., 2024; De et al., 2023; Ahmed et al., 2022). Subsequently, these chemokines and cytokines stimulate damaged melanocytes (MCs) to release their antigens, which are subsequently engulfed by antigen-presenting cells, such as dendritic cells, and presented to CD8 + T cells through MHC class I molecules (Song et al., 2024). This leads to the recruitment of melanocyte-specific CD8 + T cells that secrete interferon-gamma (IFN-*γ*), establishing a positive feedback loop. This cascade ultimately results in the targeted attack of autoantibodies on melanocytes, increased infiltration and cytotoxicity of CD8 + T cells, and upregulation of immune regulatory factors, such as IL-15-mediated activation of the JAK–STAT signaling pathway. Consequently, an adaptive immune response develops in the epidermis of vitiligo patients. Consequently, MCs dysfunction and apoptosis ensue. The unrestrained attack on MCs is attributed to compromised regulatory T cell (Tregs) activity. Memory T cells (TRMs) establish residence in lesional skin, impeding repigmentation and promoting disease relapses (Riding and Harris, 2019). The inflammatory cytokine IFN-γplays a pivotal role in this pathological process. Although IFN-γmay alter the composition and function of the gut microbiome (Yue et al., 2021), its effects are not yet fully understood. (The pathogenesis of vitiligo is shown in Figure 1).

## 2 The main metabolic products of the gut microbiota and their targets of action

### 2.1 Changes in gut microbial communities in patients with vitiligo

Metabolomic analyses of vitiligo patients have uncovered alterations in the diversity within their gut microbial communities. A trend toward diminished alpha diversity was observed in vitiligo patients, coupled with an elevated Firmicutes-to-Bacteroidetes ratio (Luan et al., 2023; Bzioueche et al., 2021). However, there is also a study that indicates a higher alpha diversity among patients with vitiligo (Ni et al., 2020), which may be attributed to the relatively small sample size of individuals included in these studies. In these studies, it was found that the proportion of Actinobacteria and Pseudomonadales increased among patients with vitiligo. This can be partially explained by the protective role of Pseudomonadales against the loss of skin pigmentation. After the application of antibiotics, there was an increase in the levels of Pseudomonadales species and a decrease in the level of depigmentation compared to mice that were not exposed (Dellacecca et al., 2020).

### 2.2 Major metabolites of gut microbiota

**Figure 2:**
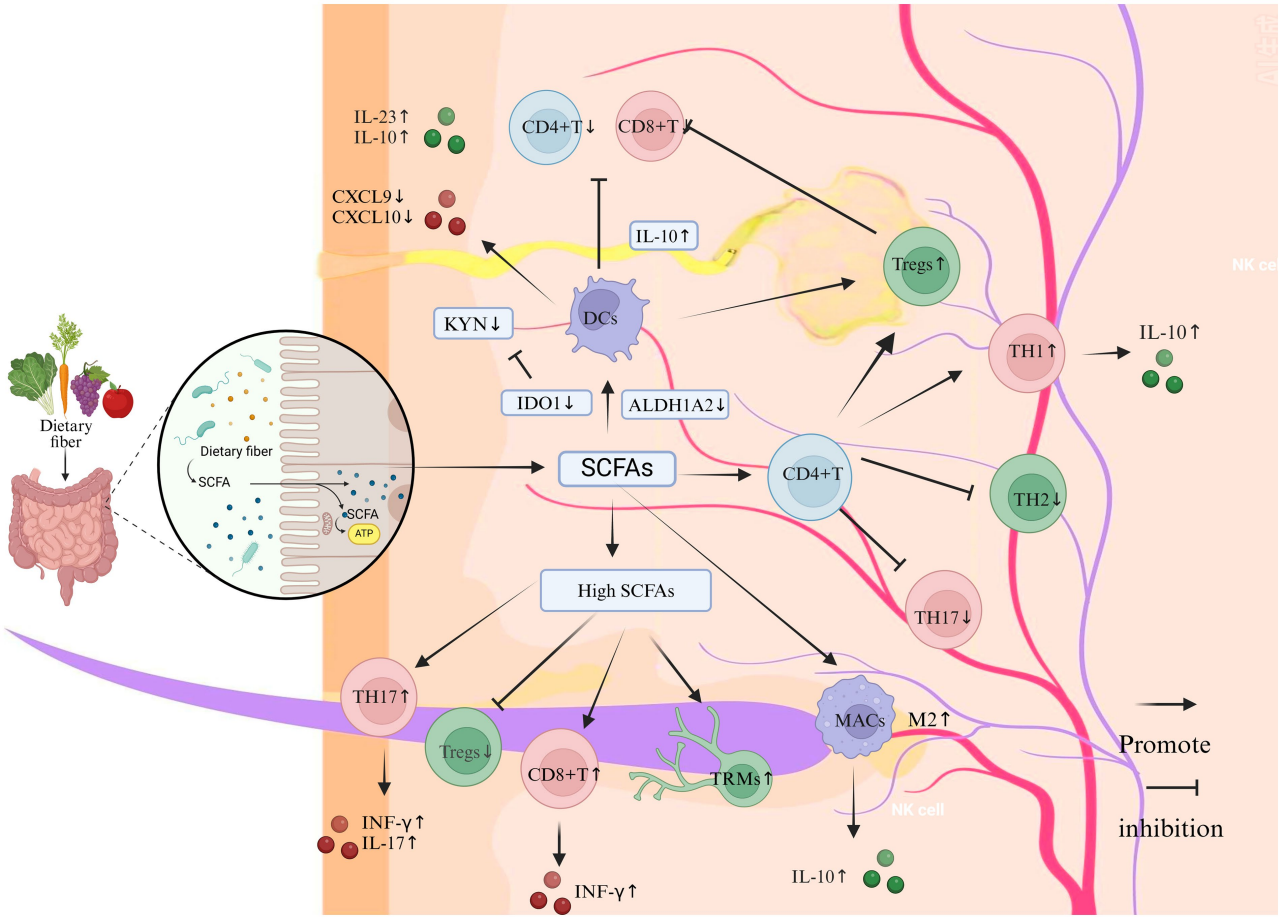
The impact of SCFAs on the immune system and inflammation in vitiligo (Created in https://BioRender.com). Dietary fiber-derived SCFAs (e.g., acetate, propionate, butyrate), generated through gut microbial fermentation, modulate cutaneous immune homeostasis via the gut-skin axis. At physiological concentrations, SCFAs enhance Tregs proliferation and potentiate their immunosuppressive capacity to restrain CD8 + T cell cytotoxicity and Th17-mediated inflammation. Mechanistically, SCFA-primed Tregs upregulate anti-inflammatory IL-10 secretion, which suppresses pro-inflammatory cytokines including IFN-γ and IL-17. Concurrently, SCFAs inhibit DCs maturation and downregulate the production of T cell-recruiting chemokines CXCL9 and CXCL10. Furthermore, SCFAs promote M2 macrophage polarization with concomitant IL-10 release, thereby facilitating tissue repair while suppressing inflammatory cascades. In contrast, supraphysiological SCFA levels exhibit paradoxical immunostimulatory effects: Th1/Th17 Polarization: Augment differentiation of Th1 and Th17 cells, elevating IFN-γ and IL-17 production. Keratinocyte Activation: Stimulate keratinocytes to release pro-inflammatory chemokines (CXCL9/CXCL10). CD8 + T Cell Recruitment: Enhance cutaneous infiltration of cytotoxic CD8 + T lymphocytes. Tissue-resident memory T cell maintenance: Sustain survival of autoreactive Trm cells in lesional skin.

**Figure 3:**
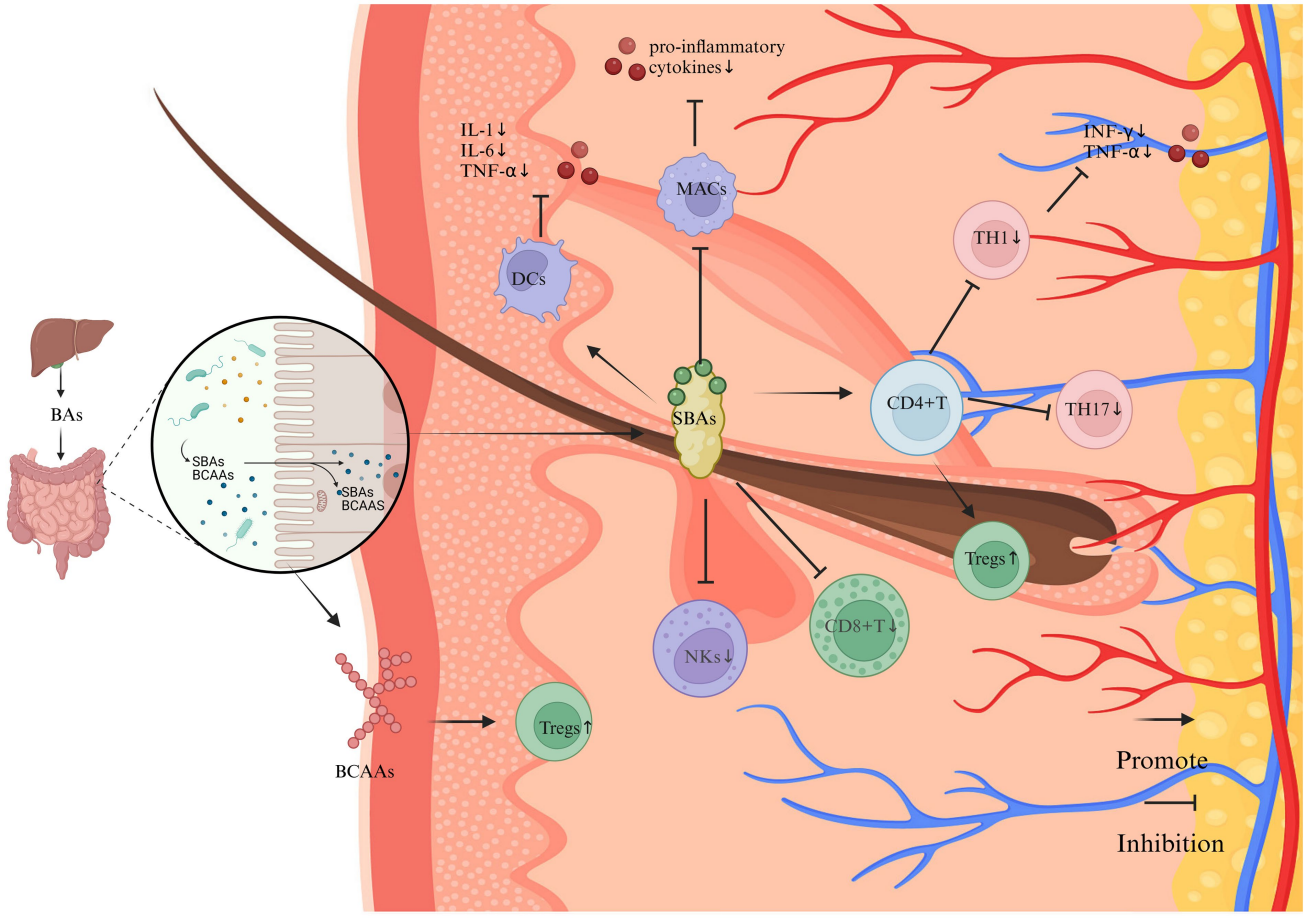
The impact of SBAs and BCAAs on the immune system and inflammation in vitiligo (Created in https://BioRender.com). SBAs, synthesized through microbial metabolism of hepatically derived primary bile acids, exhibit predominant anti-inflammatory properties through multifaceted immunomodulatory mechanisms. Key regulatory pathways include: T Cell Homeostasis: SBAs enhance Tregs differentiation via the gut-immune axis, while suppressing the polarization of pro-inflammatory Th17 and Th1 lineages. This Tregs/Th17 imbalance attenuates IFN-γ-mediated immune responses and downregulates TNF-α expression. Cytotoxic Cell Suppression: SBAs signaling inhibits the cytotoxic activity of CD8 + T lymphocytes and natural killer (NK) cells, thereby reducing melanocyte targeting. Innate Immune Regulation: Dendritic cells: Suppress pro-inflammatory cytokine secretion. Macrophages: Downregulate IL-1β, IL-6, and TNF-α production through a TGR5-dependent mechanism. Synergistically ameliorates inflammatory microenvironments in cutaneous tissues. BCAAs demonstrate complementary immunoregulatory effects, potentially augmenting Treg populations through analogous gut-immune crosstalk.

**Figure 4:**
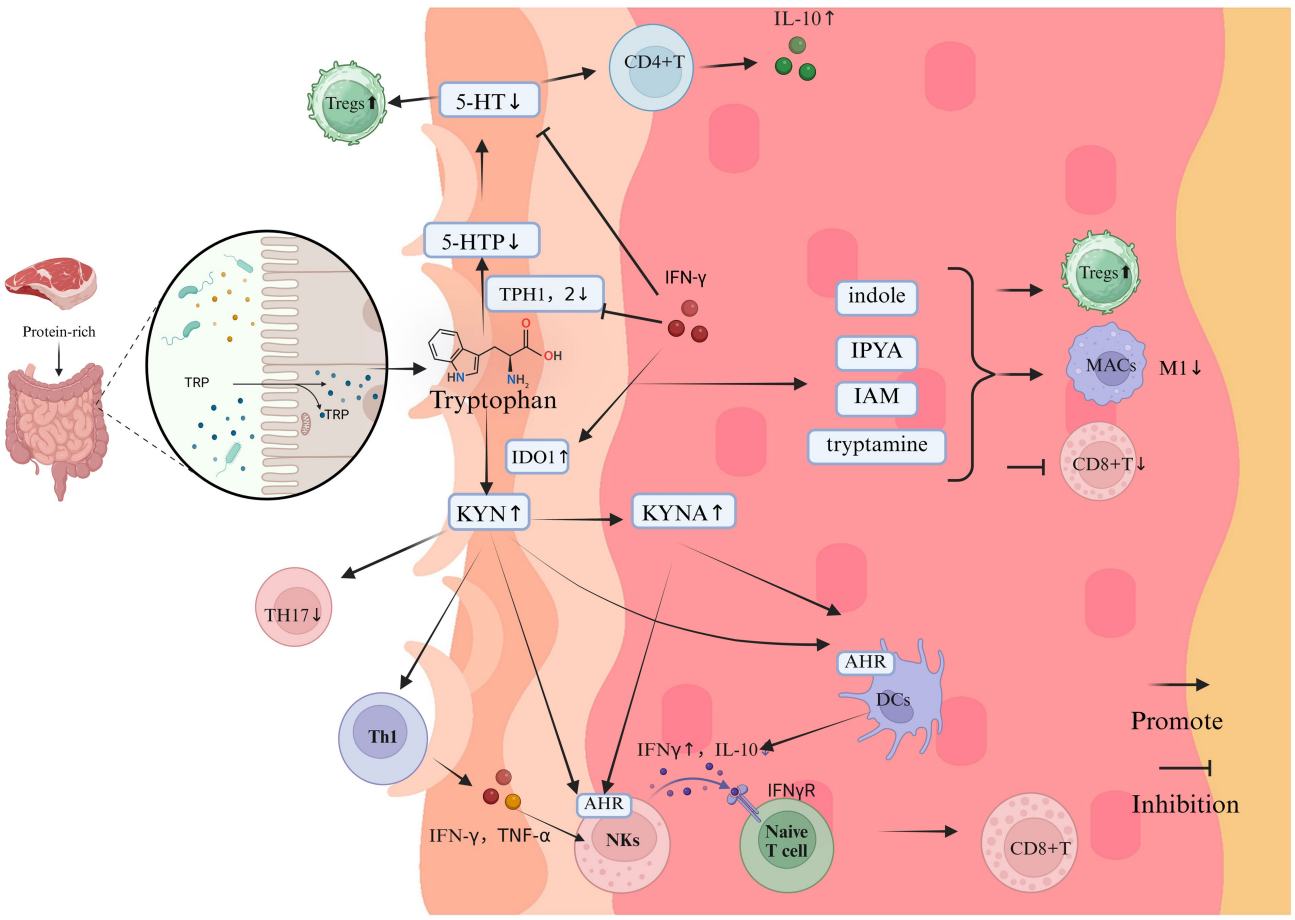
The impact of Trp on the immune system and inflammation in vitiligo (Created in https://BioRender.com). Tryptophan, derived from protein-rich diets, undergoes gut microbiota-mediated metabolism primarily through three pathways: the KYN axis, 5-HT pathway, and indole derivative biosynthesis. In vitiligo pathogenesis, dysregulation of these metabolic cascades manifests as:1. KYN Pathway Hyperactivation: Pathological Accumulation: Elevated KYN and its derivatives; Immunopathological Mechanisms: AhR Signaling: KYN activates AhR signaling in keratinocytes, triggering CXCL10 overexpression that recruits cytotoxic CD8 + T cells to melanocyte-rich areas; IFN-γ Feedback Loop: KYN-stimulated NKs release IFN-γ, which further amplifies KYN biosynthesis and melanocyte destruction via IDO1 upregulation; Anti-Inflammatory Suppression: KYNA accumulation inhibits IL-10 production, exacerbating pro-inflammatory polarization. 2. 5-HT pathway disruption. Enzymatic Inhibition: IFN-γ downregulates TPH isoforms TPH1/TPH2, reducing cutaneous 5-HT bioavailability. Melanogenic Dysregulation: Depleted 5-HT disrupts its dual modulation of melanogenesis through 5-HT1A/1B and 5-HT7 receptor signaling. 3. indole metabolite depletion. immunological consequences: Reduced microbiota-derived indoles (e.g., indole-3-aldehyde, indoleacetic acid) impair their native anti-inflammatory functions; Promotes CD8 + T cell hyperactivation and M1 macrophage polarization; Suppresses Treg differentiation via PPAR-γ/STAT3 signaling axis dysregulation.

SCFAs primarily comprise acetate (C2), propionate (C3), and butyrate (C4, 35). Specific subsets of anaerobic bacteria, in particular members of the Clostridium, Eubacterium and Butyrivibrio genera, produce SCFAs at high levels in the intestine (Singh et al., 2023). SCFAs can be transported from the gut to the skin via the bloodstream, and their concentration in the blood is sufficient to affect immune cells in distant organs, thereby modulating systemic immune responses (Liu et al., 2023). SBAs consist of deoxycholic acid (DCA), lithocholic acid (LCA), ursodeoxycholic acid, and related derivatives (Guzior and Quinn, 2021). SBAs are primarily transformed by bacterial genera including Clostridium, Enterococcus, Bifidobacterium, Lactobacillus, and Bacteroides (Cai et al., 2022). These bile acids are initially synthesized in the liver and transported to the intestine, where they undergo transformation mediated by the gut microbiota (Shapiro et al., 2018). Trp is an essential amino acid for humans and serves as an excellent substrate for various metabolic transformations within the body (Alkhalaf and Ryan, 2015). Tryptophan is primarily synthesized and metabolized through the action of various bacteria including Clostridium, Peptostreptococcus, Lactobacillus, *Escherichia coli* (*E. coli*), and Bifidobacterium (Roager and Licht, 2018). Trp is metabolized by gut microbiota through various pathways, including the kynurenine (KYN) pathway, the serotonin pathway (primarily 5-hydroxytryptamine (5-HT)), and the pathway involving indole and its derivatives. Metabolomic evidence suggests that vitiligo patients experience Trp depletion and enhanced KYN pathway metabolism, resulting in systemic accumulation of kynurenic acid (KYNA) and diminished synthesis of 5-HT and indoles (Singh et al., 2017). The potential influence of these metabolic products on vitiligo pathogenesis is depicted in Figures 2–4.

### 2.3 Targets of action of major metabolites of gut microbiota

SCFAs, such as acetate, propionate, and butyrate, signal through surface-expressed free fatty acid receptors and G protein-coupled receptors (GPCRs), including GPR41, GPR43, and GPR109A. These receptors are found on epithelial cells, adipose tissue, and various immune cells, including neutrophils, dendritic cells, macrophages, and lymphocytes, such as T cells (Sun et al., 2017). The differential expression of these GPCRs across various tissues, along with their unique affinities for specific SCFAs, could explain the differences in immune and inflammatory responses mediated by different SCFAs. In humans, GPR43 has the strongest affinity for acetate and propionate, followed by butyrate, while GPR109a has the strongest affinity for butyrate (He et al., 2020). Additionally, butyrate and propionate modulate gene expression by inhibiting histone deacetylase (HDAC) activity (Mann et al., 2024). Modulation of the immune system occurs primarily through the gut microbiota. However, commensal skin microbiota are essential for the maintenance of the skin immune homeostasis (Belkaid and Tamoutounour, 2016). In the skin, SCFAs such as propionate stimulate the production of pro-inflammatory cytokines following TLR activation (Sanford et al., 2016), which drives dendritic cell maturation and T cell proliferation (Sawada et al., 2021). Notably, butyrate exhibits an anti-inflammatory effect in the skin by reducing contact hypersensitivity and promoting the expansion of Tregs (Schwarz et al., 2017), highlighting the necessity of distinguishing between individual SCFAs. SBAs interact with a variety of receptors, including nuclear receptors—farnesoid X receptor (FXR), liver-X-receptor (LXR), pregnane X receptor (PXR), vitamin D receptor (VDR), retinoid-related orphan receptor (ROR*γ*t), and constitutive androstane receptor (CAR)—as well as membrane receptors such as G-protein bile acid receptor 1 (GPBAR1 or TGR5), sphingosine-1-phosphate receptor 2 (S1PR2), muscarinic cholinergic receptors 2 and 3 (CHRM2 and CHRM3), and MAS-related G-protein-coupled receptor family member X4 (MRGPRX4) (Biagioli et al., 2021). The aryl hydrocarbon receptor (AHR) binds to several key metabolites of Trp metabolism (Seo and Kwon, 2023). Abnormal Trp metabolism, leading to immune imbalance, is thought to be linked to AHR dysregulation (Fang et al., 2022; Zhuang et al., 2023). Reduced expression of the AHR has been associated with the progression of unstable vitiligo (Liu et al., 2021). IAId, a Trp metabolite derived from skin microbiota, has been shown to suppress skin inflammation in AD patients, underscoring the significant role of skin microbiota in the development of skin diseases (Yu et al., 2019).

## 3 The impact on T cells

### 3.1 The impact on CD4 + T cells

Upon activation, naive CD4 + T cells differentiate into various effector subsets, including Tregs, T helper 1 (Th1) cells, and T helper 2 (Th2) cells, and T helper 17 (Th17) cells (Yang et al., 2020). Th1 cells produce cytokines such as IFN-*γ* and tumor necrosis factor-alpha (TNF-*α*), which enhance the functionality of antigen-presenting DCs. Th2 cells contribute primarily to humoral immunity by producing cytokines such as IL-4, IL-5, IL-10, and IL-13 (Cenerenti et al., 2022). Th17 cells release cytokines including IL-6, IL-17, IL-21, IL-22, and TNF-α (Belpaire et al., 2022). Elevated levels of IL-17 and IL-23 in the peripheral blood of vitiligo patients implicate Th17 cells in the disease’s pathogenesis (Singh et al., 2016; Bhardwaj et al., 2020). Tregs are crucial in maintaining immune homeostasis and preventing excessive immune responses and autoimmune diseases (Mukhatayev et al., 2020). In vitiligo patients, Tregs are functionally impaired, and the Th1-skewed inflammatory microenvironment in the serum is implicated in the generation of Th1-like Tregs (Chen et al., 2022). These Th1-like Tregs exhibit a markedly diminished inhibitory effect on the proliferation and activation of CD8 + T cells. Enhanced activity of Tregs suppresses inflammation in the depigmented regions of vitiligo and inhibits CD8 + T cell-mediated attacks on melanocytes (Jin et al., 2024). Overall, a significant increase in CD4 + and CD8 + T cells, along with a marked reduction in Forkhead box P3 (Foxp3)-expressing Tregs, was observed in the marginal skin of both stable and active vitiligo cases (Abdallah et al., 2014).

TH1 cells secrete the cytokine IFN-*γ*, activating the JAK/STAT pathway, which induces keratinocytes to produce the chemokines CXCL9 and CXCL10 and promotes autoimmune destruction of melanocytes. Thus, targeting TH1 cell activity may offer a therapeutic strategy for vitiligo. Butyrate enhances Th1 cell development via upregulation of IFN-*γ* and T-bet expression, while it concurrently inhibits Th17 cell development by downregulating IL-17, Ror*α*, and Ror*γ*t expression (Chen et al., 2019). Butyrate’s influence on TH1 cell differentiation is mediated by HDAC inhibition, independent of GPR43 signaling. Butyrate’s influence on TH1 cell differentiation is mediated by HDAC inhibition, independent of GPR43 signaling. However, research also indicates that butyrate markedly decreases TH1 cell proliferation in a dose-dependent fashion (Kibbie et al., 2021). Butyrate can increase IL-10 production in TH1 cells (Sun et al., 2018). IL-10, an immunosuppressive cytokine, helps modulate TH1 cell-mediated immunity, preventing excessive inflammation and contributing to immune homeostasis. Reduced IL-10 levels, along with increased IFN-γ, perforin, and granzyme B secretion from tissue-resident memory T cells (TRMs), lead to impaired regulatory Tregs that cannot adequately suppress TRM cytotoxicity and proliferation (Shah et al., 2024). Collectively, existing studies affirm the anti-inflammatory effects of butyrates. Physiological levels of LCA suppress human and mouse Th1 cell activation, consequently reducing TNF-α and INF-γ production (Pols et al., 2017). Excessive KYN accumulation triggers AhR overactivation, which stimulates IFN-γ production by Th1 and NK cells and upregulates indoleamine 2,3-dioxygenase 1 (IDO1) expression (Shiu et al., 2022), thereby increasing Trp consumption and the accumulation of KYN pathway metabolites (Singh et al., 2017). IFN-γ not only activates the JAK–STAT pathway but also impedes melanin synthesis in response to 5-HT. Through IFNGR1/IFNGR2, IFN-γ downregulates 5-HT receptor expression, directly influencing 5-HT-induced melanin production (Cai et al., 2019).

SCFAs promote the initial differentiation of T cells into Th17 cells in a dose-dependent manner by inducing the transcription of IL-17A, IL-17F, and IFN-γ genes, and concurrently inhibit the differentiation of Tregs (Park et al., 2015). Research indicates that oral dietary fiber can enrich key gut microbiota associated with the Th17/Treg balance to alleviate inflammation, including Ligilactobacillus, Lactobacillus, Bacteroides, and Akkermansia. Concurrently, levels of microbial metabolites such as SCFAs and BAs are significantly increased (Yuan et al., 2024; Kim, 2021). Pentanoateis also a type of short-chain fatty acid that can effectively inhibit the proliferation of Th17 lymphocytes and the production of IL-17A (Luu et al., 2019). Secondary bile acids, 3-oxoLCA and isoLCA, are products of gut bacterial metabolism of lithocholic acid and exert a considerable influence on T cell differentiation and function. These metabolites inhibit TH17 cell differentiation by antagonizing the retinoic acid receptor-related orphan nuclear receptor gamma t (ROR*γ*t), a pivotal transcription factor in the promotion of TH17 cells (Hang et al., 2020; Paik et al., 2022). Kynurenine inhibits the activity of RORyt, which promotes the differentiation of pro-inflammatory Th17 cells (Riaz et al., 2022).

The investigation, a meta-analysis of 1,223 individuals with vitiligo and 1,109 control participants, revealed a significantly lower prevalence of Treg cells in the vitiligo cohort. Additionally, FOXP3 was markedly decreased in both the blood and skin tissues of patients with vitiligo (Giri et al., 2022). The reduction of proteins such as FOXP3 has been proven to be closely related to the progression of vitiligo (Abdallah et al., 2014; Giri et al., 2020). SCFAs promote the differentiation and function of Tregs by activating GPCRs and inhibiting the activity of HDAC. The enrichment of the TGF-*β*-based protocol with butyrate or propionate potentiated the *in vitro* differentiation of human naïve CD4 non-Tregs towards iTregs and augmented the suppressive capacity of the latter (Hu et al., 2022). Additionally, treatment of naive T cells with butyrate under Treg-cell-polarizing conditions enhanced histone H3 acetylation in the promoter and conserved non-coding sequence regions of the Foxp3 locus (Papaccio et al., 2024). The administration of 3-oxoLCA and isoalloLCA to mice reduced TH17 cell differentiation and increased Treg cell differentiation, respectively (Hang et al., 2020; Paik et al., 2022). SBAs induce ROR*γ*+ Tregs generation via the vitamin D receptor, essential for immune homeostasis 5-HT favors the expansion of FoxP3 Tregs and increased IL-10 production by CD4 T cells (Sacramento et al., 2018).

### 3.2 The impact on CD8 + T cells

CD8 + T cells are involved in the cytotoxicity against MCs (Fukuda, 2022), and the presence of CD8 + tissue-resident memory T cells may play a crucial role in disease recurrence (Shah et al., 2021). In individuals with vitiligo, the chemokines CXCL9 and CXCL10, predominantly secreted by keratinocytes, mediate the recruitment of CD8 + T cells to the sites of lesions. CD8 + T cells in vitiligo lesions display a hyperactive phenotype. The IFN-γ they secrete activates the JAK–STAT signaling pathway in keratinocytes, leading to the expression of chemokines CXCL9 and CXCL10. This chemokine production subsequently recruits additional CD8 + T cells to the skin, thereby establishing a positive feedback loop (Frisoli et al., 2020). Interestingly, under normal conditions, SCFAs favor IL-10-mediated immune tolerance. However, during active immune responses, SCFAs help generate the effector T cells required to clear pathogens. This can also be applied to CD8 + T cells. Specifically, SCFAs at low concentrations induce Treg differentiation, whereas at higher concentrations, they enhance CD8 + T cell recruitment and IFN-*γ* expression in a dose-dependent manner (Luu et al., 2018). Following bacterial infection, increased peripheral blood acetate enhances glycolysis-driven rapid recall responses in memory CD8 + T cells (Balmer et al., 2016). Moreover, butyrate enhances the memory T-cell response upon antigen re-encounter (Bachem et al., 2019). DCA targets plasma membrane Ca2 + ATPase to inhibit the activated T cell signaling pathway (Cong et al., 2024). Bovine bile acid diminishes the number and impairs the function of CD8 + T cells and NK cell, and impairs their effector functions (Xun et al., 2021). In patients with vitiligo, excessive accumulation of KYNA may induce the expression of CXCL10 in keratinocytes through activation of the AhR pathway, which contributes to the recruitment of CD8 + T cells at the site of lesions (Chen et al., 2024). In summary, physiological levels of SCFAs are essential for maintaining immune homeostasis. Elevated SCFAs concentrations, however, enhance the recruitment of CD8 + T cells. Previous discussions suggest that individuals with vitiligo may experience a deficiency in SCFAs, which may have led to the absence of the anti-inflammatory effects of SCFAs. And SBAs inhibit CD8 + T cell activity. Additionally, the abnormal accumulation of KYN observed in vitiligo patients facilitates CD8 + T cell recruitment, further exacerbating the condition.

## 4 The impact on dendritic cells

Dendritic cells, the primary antigen-presenting cells in the skin, play a pivotal role in mediating the interaction between innate and adaptive immune responses (Singh et al., 2021). DCs are implicated in the pathogenesis of vitiligo via antigen presentation, immune system activation, and pro-inflammatory cytokine production (Migayron et al., 2020; Srivastava et al., 2021; Singh et al., 2021). Butyrate can affect the differentiation of DCs generated from human monocytes and can inhibit T cell proliferation. Butyrate substantially down-regulates the expression of CD80, CD83, and MHC class II molecules; increases endocytic capability; reduces allostimulatory abilities; promotes IL-10 production; and inhibits IL-12 and IFN-*γ* production (Liu et al., 2012). Propionate and butyrate strongly modulated gene expression in both immature and mature human monocyte-derived DCs and diminished the production of specific DCs chemokines, such as CXCL9 and CXCL10 (Nastasi et al., 2015). Research found that DCs exposed to acetate express the immunosuppressive enzymes IDO1 and aldehyde dehydrogenase 1A2 (Aldh1A2), promote conversion of naive T-cells into immunosuppressive FoxP3(+) Tregs and suppress conversion of naive T-cells into pro-inflammatory IFN-γ-producing cells (Gurav et al., 2015). IDO1, the rate-limiting enzyme in the kynurenine pathway, the excessive activation of the KYN pathway can diminish tyrosinase activity in vitiligo-affected areas, inhibit tyrosinase expression in MCs and KCs co-cultures, and reduce melanosome numbers in the 3D human skin reconstruct model (Ferreira Branquinho et al., 2022). This complexity suggests a multifaceted interplay between gut microbiota-derived metabolites and vitiligo. DCA suppresses the expression of pro-inflammatory cytokines IL-1, IL-6, and TNF-αin bacterial lipopolysaccharide-stimulated DCs (Hu et al., 2021). Additionally, isoDCA constrains FXR activity in DCs, conferring an anti-inflammatory phenotype (Campbell et al., 2020). In summary, SCFAs attenuate the induction of autoimmunity and diminish the secretion of pro-inflammatory cytokines in DCs. Similarly, SBAs inhibit the release of pro-inflammatory cytokines from DCs. Furthermore, the deficiency of acetate may contribute to the upregulation of IDO1 in the skin, leading to an increased local depletion of trp.

## 5 The impact on macrophages

Macrophages and the macrophage migration inhibitory Factor (MIF) they secrete are associated with the severity of vitiligo. Downregulation of MIF can inhibit the activation and proliferation of CD8 + T cells in the lymph nodes of mice with vitiligo, and this effect extends to the CD8 T cells in the peripheral blood mononuclear cells of patients with vitiligo (Chen et al., 2023). Inflammatory macrophages (M1) predominantly participate in proinflammatory responses, while tissue-resident macrophages (M2) are primarily engaged in immunosuppressive activities. During active vitiligo, there is a notable increase in the infiltration of M1 macrophages in the bloodstream, which triggers a T-helper 1 (Th1) or T-helper 17 (Th17)-mediated immune response. This response exacerbates the destruction of melanocytes and leads to increased depigmentation. Conversely, the proportion of M2 macrophages remains comparable to that found in healthy tissue (Sain et al., 2024). SCFAs inhibit pro-inflammatory cytokines through HDAC activity suppression and also promote anti-inflammatory IL-10 expression in macrophages (Liu et al., 2012). Butyrate exerts anti-inflammatory effects by enhancing STAT6 signaling and inhibiting HDAC1, thereby promoting M2 macrophage polarization (Ji et al., 2016). SBAs inhibit the release of pro-inflammatory factors in macrophages induced by bacterial lipopolysaccharides in a TGR5-dependent manner (Haselow et al., 2013). Reduced Trp intake promotes M1-type macrophage polarization and facilitates CD8 + T cell accumulation, whereas restoring Trp intake reverses this effect (Jiang et al., 2024). GPR35^+^ macrophages constitute a pro-inflammatory subset found in the small intestine. These cells facilitate the Th17 immune response through the KYNA-GPR35 signaling pathway and are involved in the pathogenesis of autoimmune diseases. KYNA modulates the recruitment and aggregation of GPR35^+^ macrophages (Miyamoto et al., 2023). While the exact mechanisms through which gut microbiota-derived metabolites influence macrophages in vitiligo remain to be fully elucidated, the modulation of macrophage polarization represents a potential mechanism for ameliorating vitiligo lesions.

## 6 The direct effects of gut microbiota metabolites on vitiligo and melanocytes

Research observed a reduction in the abundance of bacterial taxa typically associated with a healthy gut microbiome, as well as a decrease in SCFAs-producing taxa in individuals with vitiligo (Kumar et al., 2024). Butyrate plays a crucial role in maintaining the integrity of the intestinal barrier and exhibits anti-inflammatory and immune-regulatory properties in autoimmune diseases. However, its efficacy can be affected by factors such as concentration, site of action, and the physiological status of the host (Kumar et al., 2024; Gerunova et al., 2024). Studies on neonatal melanocytes have shown that butyrate is cytotoxic to primary melanocytes at concentrations above 1 mM (Du et al., 2022). At lower, non-toxic concentrations (0.5 and 1 mM), however, butyrate significantly enhances melanocyte differentiation, resulting in melanosome formation and increased pigment deposition (Goenka, 2024). Topical application of butyrate, either alone or in combination with *S. epidermidis* and glycerol, significantly reduced UVB-induced IL-6 production (Keshari et al., 2019). Propionate effectively reduces melanin content in melanocytes, and treatment with 4 mM propionate significantly inhibits tyrosinase activity without affecting cell proliferation, indicating that propionate’s suppression of melanogenesis occurs via downregulation of tyrosinase gene expression (Kao et al., 2021). Although the aforementioned studies appear to contradict the anti-inflammatory activity of SCFAs, it is important to note that SCFA levels may be reduced in vitiligo patients, potentially diminishing their anti-inflammatory and high-dose cytotoxic effects. Additionally, differential GPCRs expression in tissues and their specific affinity for SCFAs must be considered. This evidence collectively suggests that SCFAs likely exert pleiotropic physiological roles through multiple pathways, with their net effects potentially modulated by concentration gradients, tissue-specific distribution, and host metabolic status.

Research indicates that Ursodeoxycholic Acid (UDCA, a secondary bile acid generated by the gut microbiota through modifications such as hydroxylation of primary bile acids) mitigates the cellular impact of UV light exposure on human skin by diminishing intracellular oxidative stress and cutaneous inflammation. In this experiment, UDCA has been shown to reduce melanin content in normal human melanocytes (Moon et al., 2021). However, this study was based on experiments constructed using an aging skin model, which is not consistent with the pathogenesis of vitiligo. Moreover, UCDA has demonstrated anti-inflammatory and antioxidant effects. Therefore, the role of UCDA in vitiligo requires further exploration.

At a concentration of 5 mM, KYN significantly inhibited DNA synthesis in melanocytes (Walczak et al., 2020). Further research has shown that KYN, originating from sources such as microbial metabolism and fibroblast production, inhibits DNA synthesis and significantly reduces metabolic activity in primary human melanocytes (Walczak et al., 2023). Excessive accumulation of kynurenine can also impair tyrosinase activity in vitiligo-affected areas, downregulate tyrosinase expression in melanocyte and keratinocyte co-cultures, and decrease melanosomes in 3D human skin models (Ferreira Branquinho et al., 2022). As previously mentioned, vitiligo patients exhibit elevated blood levels of kynurenine aminotransferase, leading to kynurenine pathway deviation and systemic accumulation of KYNA (Singh et al., 2017). Consequently, excessive KYN accumulation in vitiligo patients may damage melanocytes and contribute to the pathophysiology of the disease. Oxidative stress can deplete epidermal Trp, leading to lower serotonin and melatonin levels (Schallreuter et al., 2012). *In vitro* studies show that melanophores in lower vertebrates exhibit dose-dependent pigmentation in response to 5-HT1 and 5-HT2 receptor agonists, while 5-HT3 and 5-HT4 receptor agonists induce dose-dependent pigment aggregation (Ali et al., 2012; Yue et al., 2022; Wu et al., 2014). The serotonin/5-HT7 receptor mediates an adaptive response that enhances pigmentation under environmental stress through various signaling pathways, including cAMP-PKA-MAPK, Rab27a/RhoA, and PI3K/AKT (Tang et al., 2023). Emotional stress can reduce skin serotonin levels, thereby affecting melanin production (Liao et al., 2017). Consequently, fluoxetine, a serotonin reuptake inhibitor, has been shown to effectively treat pigment loss disorders (Slominski et al., 2012). Indole derivatives, as endogenous ligands of the AHR, activate AHR signaling and alleviate psoriasis and certain dermatitis conditions (Uberoi et al., 2021; Furue et al., 2019). However, direct evidence of their effect on vitiligo is currently lacking. The details of the above study can be found in Table 1.

**Table 1:** The direct effects of gut microbiota metabolites on vitiligo and melanocytes.

Years	Metabolites	Research models or objects	Research results	Author
2024	Butyrate	Primary human MCS	1. Butyrate shows cytotoxicity at high concentrations (>1 mM) and promotes differentiation at low concentrations. 2. It does not affect MITF levels but stimulates tyrosinase synthesis while inhibiting its activity, with no impact on melanin. 3. Butyrate also induces IL-6 without affecting oxidative stress.	Shilpi Goenka
2019	Butyrate	Dorsal skin of mice	1. UVB exposure on mice skin increased IL-6 production. 2. Butyric acid or *S. epidermidis* with glycerol reduced UVB-induced IL-6. Knocking down FFAR2 in mouse skin blocked this probiotic effect.	Keshari S
2021	Propionate	DOPA-positive MCs AND mice ear skin tissue	1. Propionate from Pluronic F68 fermentation of Cutibacterium acnes was the most abundant fatty acid and reduced DOPA-positive melanocytes by inhibiting tyrosinase activity via FFAR2 binding. 2. Propionate at 4 mM did not affect melanocyte proliferation, making it a safe and effective treatment for hyperpigmentation.	Kao H. J.
2021	UCDA	Senescent skin	1. UDCA reduced UV-induced ROS, SASP, and pro-inflammatory cytokines. 2. UDCA decreased melanogenesis in human melanocytes co-cultured with skin cells.	Moon I. J.
2020	KYN	MCs and melanoma A375	1. KYN, KYNA and FICZ in higher concentrations inhibited the protein level of AhR but did not affect the gene expression.	Walczak K.
2023	KYN	MCs	1. KYN inhibited MCs cell metabolism by reducing cyclin D1 and CDK4 levels via the AhR pathway.	Walczak K.
2012	5-HT	Amphibian pigment cells	1. In vitro studies show that melanophores in lower vertebrates exhibit dose-dependent pigmentation in response to 5-HT1 and 5-HT2 receptor agonists, while 5-HT3 and 5-HT4 receptor agonists induce dose-dependent pigment aggregatio	Ali S. A.
2022	5-HT2R	Mouse melanoma cell line B16F10, human skin, and zebrafish embryos	1. HTR2A antagonists reduced serotonin-induced melanogenesis in mouse melanoma cells and zebrafish embryos. 2. HTR2A agonists increased melanin synthesis and transfer in B16F10 cells, human skin tissue, and zebrafish embryos.	Yue Y.
2014	5-HT1A/1B	C57BL/6 mouse skin	1. The serotoninergic system plays an important role in the regulation of stress-induced depigmentation, which can be mediated by 5-HT1A/1B receptors	Wu H. L.
2023	5-HT7R	Human skin cells, human tissue, mice, and zebrafish models	1.5-HT7R selective agonist, LP-12, had been demonstrated to enhance melanin production, dendrite growth, and chemotactic motility in B16F10 cells, normal human melanocytes, and zebrafish.	Tang H. H.
2017	5-HT	Mouse skin	1. Stress altered hippocampal morphology, reduced brain and skin 5-HT levels, and downregulated 5-HT1A receptors in the hippocampal CA1 region and skin. 2. Stress caused melanocyte damage and inhibited keratinocyte proliferation in hair follicles, leading to pigment loss.	Liao S.

## 7 Treatment based on gut microbiota metabolites

Diet emerges as a pivotal determinant of gut microbiota community structure and function (Zmora et al., 2019; De Angelis et al., 2019). The effects of diet on host metabolism and physiology can be mediated through the gut microbiota (Guan and Liu, 2023). Specific nutrients present in the diet can be metabolized into bacterial metabolites, thereby influencing the composition of gut microbiota. Secondly, ingestion can introduce probiotic or pathogenic bacteria from diet into the intestinal microenvironment, leading to alterations in the resident gut microbiota population and composition (Hills et al., 2019; Wilson et al., 2020). Thirdly, consuming certain diets may alter the physical environment within the gut, including the pH level, thereby potentially changing the composition of the gut microbiota. In this context, dietary intervention through the use of probiotics, prebiotics and antioxidant foods can be considered a contribution to the modulation of vitiligo (Ferreira et al., 2022).

### 7.1 The impact of diet on gut microbiota

The Mediterranean diet (Med) has been demonstrated to elevate levels of Bacteroidetes and Prevotellaceae families, thereby increasing the concentration of SCFAs (Gutiérrez-Díaz et al., 2016). In a preliminary four-day study, Indole-3-acetic acid (IAA), Indole-3-propionic acid(IPA), and Indole-3-lactic acid (ILA) were significantly increased after the Mediterranean diet and decreased after the fast-food diet (FF), whereasTrp, indole-6-carboxaldehyde, and 4-(1-piperazinyl)-1H-indole increased after FF and decreased after Med. The ratio of kynurenine to Trp was significantly decreased after FF and increased after Med, and no change in bile acids was observed in this study (Zhu et al., 2020). Currently, there is a lack of research on the Mediterranean diet as a potential adjunctive therapy for individuals with vitiligo. This gap in the literature warrants future investigation into its therapeutic efficacy.

### 7.2 The application of probiotics and prebiotics

Probiotics are live microorganisms that, when consumed in adequate amounts, confer a health benefit on the host. They are commonly found in fermented foods such as yogurt, kefir, and pickles, as well as in dietary supplements. Prebiotics, on the other hand, are indigestible food components that stimulate the growth and activity of beneficial bacteria in the gut, and are typically present in fiber-rich foods such as fruits, vegetables, and whole grains. In the past ten years, the number of commercially available topical probiotics has dramatically increased (Lee et al., 2019), and probiotics have been applied topically and orally to treat various skin disorders, such as acne, atopic dermatitis, and rosacea (França, 2021). Prebiotics or symbiotics have been demonstrated to significantly improve skin health by improving the gut microbiota and its metabolites (Al-Smadi et al., 2023).

### 7.3 Fecal microbiota transplantation

Fecal Microbiota Transplantation (FMT) entails the transfer of gut microbiota from healthy donors to patients. FMT, which is primarily used to treat intestinal diseases by reshaping and balancing the gut microbiota, also appears to be a viable approach for managing skin inflammatory diseases (Wu et al., 2024). Moreover, the application of FMT in various autoimmune disorders has demonstrated promising safety and efficacy, which lays a theoretical foundation for its potential use in the treatment of vitiligo. Patients undergoing FMT have exhibited increased diversity in gut microbiota, especially in SCFAs-producing bacteria, resulting in a significant elevation in fecal SCFAs concentration (Huang et al., 2022). Patients with alopecia areata have shown improvement after undergoing FMT therapy (Rebello et al., 2017; Xie et al., 2019), and since the pathogenesis of alopecia areata and vitiligo shares commonalities, targeting the microbiome through methods such as fecal FMT is an emerging and promising strategy. However, the precise effects and potential mechanisms of FMT in the treatment of vitiligo require further clinical research to be confirmed.

### 7.4 Other potential dietary management strategies

Additionally, a growing body of evidence suggests the role of herbal bioactive compounds and nutritional supplements in vitiligo, including *Ginkgo Biloba*, Polypodium leucotomos, Capsaicin, Curcumin, *Phyllanthus emblica* fruit extract, carotenoids, Canthaxanthin, *Nigella sativa* seed oil, Picrorhiza kurroa, and Khellin (Shakhbazova et al., 2021). A research finds that the total-fat content of the diet had a more impressive role than the specific subclasses of fats on the incidence risk of vitiligo (Derakhshandeh-Rishehri et al., 2019). Therefore, reducing fat intake may be an effective strategy for preventing the recurrence of vitiligo. Based on a mendelian randomization study, vitiligo patients are advised to avoid excessive alcohol consumption. However, moderate intake of wine, cider, beer and champagne may be acceptable. In addition, vitiligo patients should limit their intake of tea and bread. And consuming high-fibre and probiotic-rich foods may help vitiligo patients regulate their gut microbiota overall (Zhang et al., 2024).

The complexity of the pathogenesis of vitiligo and its tendency to relapse highlight the fragile immune homeostasis in patients with the condition. The gut microbiota and its metabolic byproducts have a profound and distinct impact on the host’s immune balance and inflammatory stability. While dietary interventions cannot be thought of as a standalone therapy, they still make a case for being used as adjuncts (Hadi et al., 2024). In the future, it is worth further exploration to manage vitiligo by utilizing the gut-skin axis and regulating the metabolic products of the gut microbiota.

## 8 Discussion

This review is grounded in the “gut-skin axis” theory and systematically examines the regulatory effects of gut microbiota-derived metabolites on immune dysregulation and melanocyte dysfunction in vitiligo. Current evidence indicates that microbial metabolites, including SCFAs, SBAs, and Trp catabolites, significantly influence disease progression through modulation of immune cell differentiation (particularly Th17/Treg balance and CD8 + T cell cytotoxicity) and inflammatory cytokine networks. Notably, the concentration-dependent biological effects of these metabolites (e.g., low-dose butyrate promotes tissue repair while high concentrations exacerbate inflammatory responses) reveal complex microbiota-immune microenvironment interactions. Clinical observations demonstrate that vitiligo patients exhibit reduced gut microbial diversity and accumulated pro-inflammatory metabolites, which correlate with disrupted immune homeostasis, suggesting microbial metabolic dysbiosis as a potential pathogenic driver. However, discrepancies in research outcomes, particularly regarding the directionality of *α*-diversity alterations in gut microbiota, may stem from sample heterogeneity or individual variations in host-microbiota crosstalk. Furthermore, caution is warranted when extrapolating *in vitro* findings to physiological microenvironments. These mechanistic insights provide a theoretical foundation for developing novel metabolic-immunological therapeutic strategies, highlighting the potential clinical value of restoring immune equilibrium through targeted modulation of gut microbiota and their metabolic networks.

In summary, this review is based on the gut-skin axis theory and investigates the impact of gut microbiota metabolic products on vitiligo. We hope that this research will stimulate a comprehensive discussion on the interaction mechanisms between the gut microbiota and vitiligo. Future explorations are warranted to examine the potential of using dietary fiber supplementation or probiotics as intervention measures to regulate the immune dysregulation in vitiligo and promote repigmentation in the depigmented areas.

### 8.1 Future challenges

1. According to our current research, a change has been observed in the gut microbiota composition of patients with vitiligo, a phenomenon that has garnered our significant attention. Additionally, we have discovered a reduction in the production of microbial metabolic products such as SCFAs, 5-HT, and indole compounds, which may have adverse effects on the health of patients. Conversely, the accumulation of KYN is excessive and this alteration cannot be overlooked. These factors seem to collectively influence the progression of vitiligo in patients, yet to date, no comprehensive study has been able to fully explain this issue, warranting further research in the future.
2. It remains uncertain whether there is a difference in the gut microbiota between patients with stable vitiligo and those with rapidly progressing disease, which is a question that requires in-depth exploration. At the same time, we are also considering whether the dysregulation of gut microbiota metabolic products plays an important role in the onset of vitiligo during rapid progression, which is equally a direction worthy of future study.
3. Furthermore, it is worth investigating whether targeted dietary interventions, such as the supplementation of dietary fiber, could improve the clinical manifestation of vitiligo patients. This is a potential therapeutic approach that may bring new hope to those suffering from vitiligo.
